# Hare Lip

**Published:** 1869-09

**Authors:** 


Hare Lip.—Cases of hare-lip ought to be operated upon the first month; nothing is gained by waiting. If this is not done the child fails to take sufficient food, and becomes emaciated and unfit for the operation. The best position for the operator is sitting, the head of the child being held between the knees, while the rest of the body is supported by an assistant. Use a narrow scalpel, with which transfix above and cut downwards in a curved direction, that when the two curved surfaces are brought together the full edge of the lip may be thrown sufficiently down. If the alveolar process is cleft and prominent it should now be snipt through at the spot between the lateral incisor tooth and the canine, and pushed back. Having done this the lip and all the surrounding parts, including the nose, must be separated from the deeper parts, so that the lip may hang loosely, without any tension. If this is not well done there will be failure in the union. The raw edges of the wound must now be adjusted, commencing the adaptation at the lowest part. The sutures and pins are generally removed too early. (Mr. H. Walton, p. 157.)

New Operation for.—The author describes a new operation for the remedy of this deformity. The peculiar object of this operation is the preservation of the natural curves of the lip. The upper lip may be divided into a central and two lateral portions, divided by curved lines running from the nostril to the margin of the lip. The fissures of hare-lip always correspond to this division, and the cicatrices should also do so. (Dr. M. H. Collis, p. 154.)

